# A putative multi-replicon plasmid co-harboring beta-lactamase genes *bla*_KPC-2_, *bla*_CTX-M-14_ and *bla*_TEM-1_ and trimethoprim resistance gene *dfrA25* from a *Klebsiella pneumoniae* sequence type (ST) 11 strain in China

**DOI:** 10.1371/journal.pone.0171339

**Published:** 2017-02-02

**Authors:** Yu Tang, Pinghua Shen, Wei Liang, Jialin Jin, Xiaofei Jiang

**Affiliations:** 1 Department of Laboratory Medicine, Huashan Hospital, Shanghai Medical College, Fudan University, Shanghai, China; 2 Department of Laboratory Medicine, Shanghai First Maternity and Infant Hospital, Tongji University School of Medicine, Shanghai, China; 3 Department of Infectious Diseases, Huashan Hospital, Shanghai Medical College, Fudan University, Shanghai, China; Tianjin University, CHINA

## Abstract

The global emergence of *Klebsiella pneumoniae* carbapenemase (KPC)-producing *Klebsiella pneumoniae* poses a major public health threat requiring immediate and aggressive action. Some older generation antibiotics, such as trimethoprim, serve as alternatives for treatment of infections. Here, we determined the complete nucleotide sequence of plasmid pHS091147, which co-harbored the carbapenemase (*bla*_KPC-2_) and trimethoprim resistance genes (*dfrA25*) from a *Klebsiella pneumoniae* sequence type (ST) 11 clone recovered in Shanghai, China. pHS091147 had three replication genes, several plasmid-stability genes and an intact type IV secretion system gene cluster. Besides *bla*_KPC-2_ and *dfrA25*, pHS091147 carried several other resistance genes, including β-lactamase genes *bla*_TEM-1_ and *bla*_CTX-M-14_, sulphonamide resistance gene *sul1*, a quinolone resistance gene remnant (Δ*qnrB2*), and virulence associated gene *iroN*. Notably, the multidrug-resistance region was a chimeric structure composed of three subregions, which shared strong sequence homology with several plasmids previously assigned in Genbank. To our knowledge, this is the first report of the co-localization of *bla*_KPC-2_ and *dfrA25* on a novel putative multi-replicon plasmid in a *Klebsiella pneumoniae* ST11 clone.

## Introduction

A dramatic increase in the prevalence of *Klebsiella pneumoniae* carbapenemase (KPC)-producing *K*. *pneumoniae* is associated with a rise in morbidity and mortality, and poses an alarming clinical threat for hospitalized patients [1]. KPC-2 is the most common variant of the KPC enzymes. The large majority of KPC-2-producing *K*. *pneumoniae* in America and Europe belongs to sequence type (ST) 258 clone; in China, the majority belongs to the ST11 clone, which is a part of the same clonal complex as ST258 [2, 3].

Since the increase of carbapenem-resistant *Enterobacteriaceae*, which are frequently resistant to many different antibiotic substances, some older generation antibiotics (e.g., trimethoprim) have been used alternatively for treating infections [4, 5]. With a structure similar to that of folic acid, trimethoprim is a competitive inhibitor of dihydrofolate reductase [6, 7]. Bacterial resistance to trimethoprim can be inherited or acquired. The most common trimethoprim resistance mechanism involves acquisition of trimethoprim-resistant dihydrofolate reductase (*dfr*) gene [6, 7]. Until now, more than 25 different trimethoprim resistance *dfrA* genes have been identified; the majority are associated with mobile genetic elements such as plasmids, transposons or integrons [8, 9]. The gene *dfrA25* was firstly detected as a gene cassette within a class 1 integron in *Salmonella* Agona [10]. In this study, we report the coexistence of *bla*_KPC-2_ *and dfrA25* on a single putative multi-replicon plasmid obtained from an epidemic *K*. *pneumoniae* ST11 isolate recovered in China.

## Materials and methods

### Bacterial strain and plasmid

*K*. *pneumoniae* HS091147 used in this study was isolated in 2009 from a sputum sample at Huashan Hospital, Shanghai Medical College, Fudan University, China. Plasmid DNA was extracted from *K*. *pneumoniae* HS091147 (Qiagen plasmid mid kit; Qiagen, Germany) and transferred by electroporation (Micro-Pulser electroporator; Bio-Rad, USA) into *E*. *coli* DH5α. Transformants were selected on Luria-Bertani (LB) agar plates containing ampicillin (100 μg/ml) and imipenem (2μg/ml), then screened by a *bla*_KPC-2_ PCR assay. The primers targeting *bla*_KPC-2_ genes were described previously [11].

### Antibiotic susceptibility testing

The minimal inhibitory concentrations (MICs) for *K*. *pneumoniae* HS091147, its transformant and *E*. *coli* DH5α were determined using the VITEK^®^2 COMPACT AST-GN13 (bioMérieux, France). *E*. *coli* ATCC 25922 was used as the quality control strain. All susceptibility tests were repeated three times and the results were interpreted according to the breakpoints suggested by the Clinical and Laboratory Standards Institute (CLSI) [12].

### Multilocus Sequence Typing (MLST)

The sequencing types (STs) of *K*. *pneumoniae* strain HS091147 were determined by analyzing *gapA*, *infB*, *mdh*, *pgi*, *phoE*, *rpoB*, and *tonB* housekeeping genes. The results were compared with information provided in the multilocus sequence typing (MLST) databases (http://www.pasteur.fr/recherche/genopole/PF8/mlst/Kpneumoniae.html).

### Sequencing and annotation of plasmid

Complete sequencing of the plasmid pHS091147 was performed with a shotgun approach using 454 GS Junior (Roche, Basel, Switzerland). The GenBank accession number is KX236178.

## Results and discussion

### HS091147 and its *E*. *coli* DH5α transformant (T-pHS091147)

*K*. *pneumoniae* HS091147 was characterized according to the species-specific MLST schemes of the Pasteur database and found to belong to the *K*. *pneumoniae* ST11 clone. ST11 was previously determined as the prevalent clone associated with the spread of KPC in Asia (particularly in China) [2, 13], while other β-lactamases (e.g., CTX-M-type ESBLs) did not show similar epidemiological characteristics [14–16].

The MICs of *K*. *pneumoniae* HS091147 and its transformant are shown in Table 1. Notably, the original HS091147 strain was resistant to all 17 antibiotics tested (ampicillin, ampicillin/sulbactam, cefazolin, cefotetan, ceftazidime, ceftriaoxone, cefepime, aztreonam, ertapenem, imipenem, amikcin, tobramycin, ciprofloxacin, levofloxacin, nitrofurantoin, piperacillin/tazobactam and trimethoprim/sulfamethoxazole), and 7 of these (ampicillin, ampicillin/sulbactam, cefazolin, ceftriaoxone, aztreonam, imipenem, and trimethoprim/sulfamethoxazole) were fully transferable to the recipient, *E*. *coli* DH5α strain via the plasmid pHS091147. Furthermore, the transformant T-pHS091147 was intermediately resistant to ertapenem and piperacillin/tazobactam when compared to the DH5α background.

**Table 1:** Antibiotic resistance profiles for *K*. *pneumoniae* HS091147 and its transformant (T-pHS091147) in *E*. *coli* DH5α.

Antibiotics	Strain		
	HS091147	DH5α	T-pHS091147
Ampicillin	≥32 (R)	≤2 (S)	≥32 (R)
Ampicillin/Sulbactam	≥32 (R)	≤2 (S)	≥32 (R)
Cefazolin	≥64 (R)	≤4 (S)	≥64 (R)
Cefotetan	≥64 (R)	≤4 (S)	≤4 (S)
Ceftazidime	≥64 (R)	≤1 (S)	4 (S)
Ceftriaoxone	≥64 (R)	≤1 (S)	≥64 (R)
Cefepime	≥64 (R)	≤1 (S)	2 (S)
Aztreonam	≥64 (R)	≤1 (S)	16 (R)
Ertapenem	≥8 (R)	≤0.5 (S)	1 (I)
Imipenem	≥16 (R)	≤1 (S)	8 (R)
Amikcin	≥64 (R)	≤2 (S)	≤2 (S)
Tobramycin	≥16 (R)	≤1 (S)	≤1 (S)
Ciprofloxacin	≥4 (R)	≤0.25 (S)	≤0.25 (S)
Levofloxacin	≥8 (R)	≤0.25 (S)	≤0.25 (S)
Nitrofurantoin	≥512 (R)	≤16 (S)	≤16 (S)
Piperacillin/Tazobactam	≥128 (R)	≤4 (S)	64 (I)
Trimethoprim/Sulfamethoxazole	≥320 (R)	≤20 (S)	≥320 (R)

R, resistant; I, intermediate resistant; S, susceptible

### General features of plasmid pHS091147

**Fig 1:**
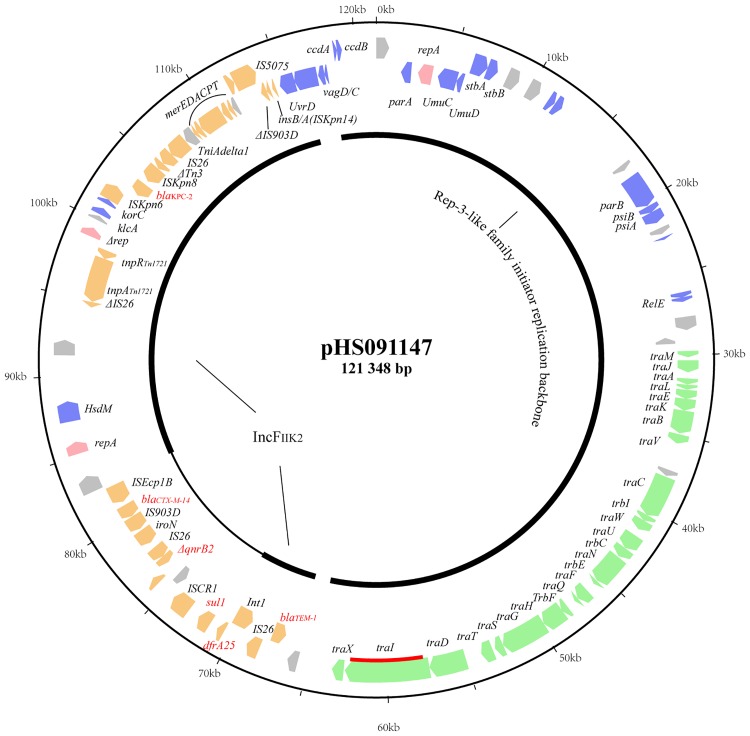
Circular map of pHS091147. Genes are color-coded dependent upon functional annotations as follows: pink, replication; blue, stability; green, propagation; orange, adaptation (the MDR region); grey, other functions and hypothetical proteins. The relaxase gene (*traI*) is indicated by the red bar. Red text highlights the resistance genes: *bla*_KPC-2_, *bla*_TEM-1_, *bla*_CTX-M-14_, *dfrA25*, *sul1 and* Δ*qnrB2*.

Plasmid pHS091147 was determined to be a circular molecule, 121,348 bp in size with an average G+C content of 52.8%. Annotation revealed 107 predicted open reading frames. One hundred and one of these frames encoded proteins homologous to proteins with known functions and assigned to other sequenced plasmids in GenBank. Using the approach by Norman *et al*. [17], pHS091147 was determined to carry genes involved in replication, stability, propagation and adaptation (Fig 1).

### Replication, stability and propagation of pHS091147

Plasmid pHS091147 contains three replication genes, one of which was truncated with loss of function (*Δrep* in Fig 1, position 98903 to 99463). The other two genes, *rep*FII_K2_ (position 85132 to 85887) and an unassigned gene (position 2795 to 3745), were located between the MDR region and the plasmid backbone, respectively. The *rep*FII_K2_ gene is frequently reported in KPC-encoding plasmids and may be the primary vehicle for the dissemination of *bla*_KPC_ [18, 19]. The unassigned gene, encoding a Rep-3-like family initiator replication protein, was only found in 28 plasmid sequences deposited in GenBank. Among these, the presence of two single-replicon plasmids, pNJST258C3 and pNJST258N3 (GenBank accession numbers: CP006925 and CP006921, respectively), suggested the likelihood that the unassigned replicons function in initiation of replication.

Genes that encode replication (*repA*), stability (e.g., *ParA*, *stbA/stbB* and *UnuC/UmuD* surrounding the *repA* gene) and propagation were located within the unassigned Rep-3-like family backbone of pHS091147. In addition, we found a full complement of conjugation machinery including a type IV secretion system (T4SS) and relaxase, which enabled mobilization [17].

**Fig 2:**
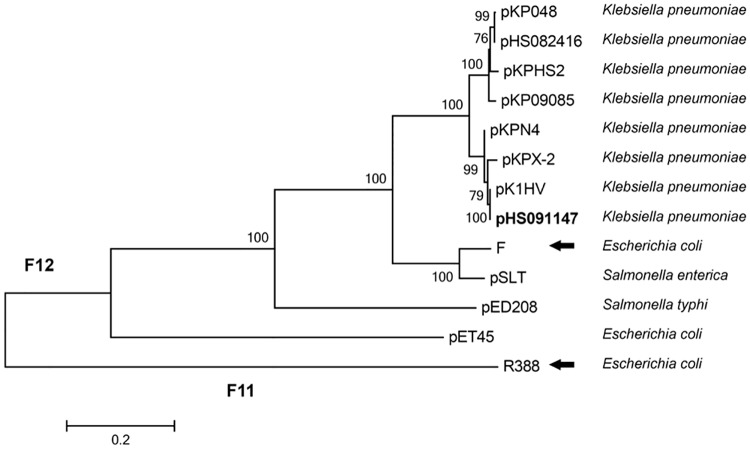
Phylogenetic analysis of plasmid-encoded relaxase homologs. Plasmid pHS091147 (in bold) and twelve other protein sequences were aligned, and the tree was generated with MEGA5 using the maximum-likelihood method. Solid black arrows point to the prototype plasmids for the MOB_F12_ and MOB_F11_ subfamilies. Other relaxase sequences of plasmids pKP048 (GenBank accession number FJ628167), pHS082416 (KF724507), pKHS2 (CP003224), pKP09085 (KF719970), pKPN4 (CP000649), pKPX-2 (AP012056), pK1HV (HF545434), F (AP001918), pSLT (AE006471), pED208 (AF411480), pET45 (CU468132) and R388 (BR000038) were obtained from GenBank.

Relaxase, the only common component in all transmissible plasmids, can be used to classify plasmids and infer phylogenetic relationships [20–22]. Plasmids can be classified in six mobility (MOB) families: MOB_F_, MOB_P_, MOB_Q_, MOB_H_, MOB_C_, and MOB_V_, according to the relaxase sequence. MOB_F_, a well-characterized family, contains several subfamilies [20–22]. Phylogenetic analysis showed that the relaxase encoded by pHS091147 was grouped in the F12 subfamily (Fig 2). Comparison of the pHS091147 relaxase protein sequence revealed 79% identity with the sequence found in F (GenBank accession number: AP001918), the prototype plasmid for the F12 subfamily (IncF12), and 100% identity with that of pK1HV (GenBank accession number: HF545434), a *K*. *pneumoniae* multidrug resistance plasmid harboring *qnrS1* isolated in Vietnam.

### The adaptation region is highly mosaic-like

**Fig 3:**
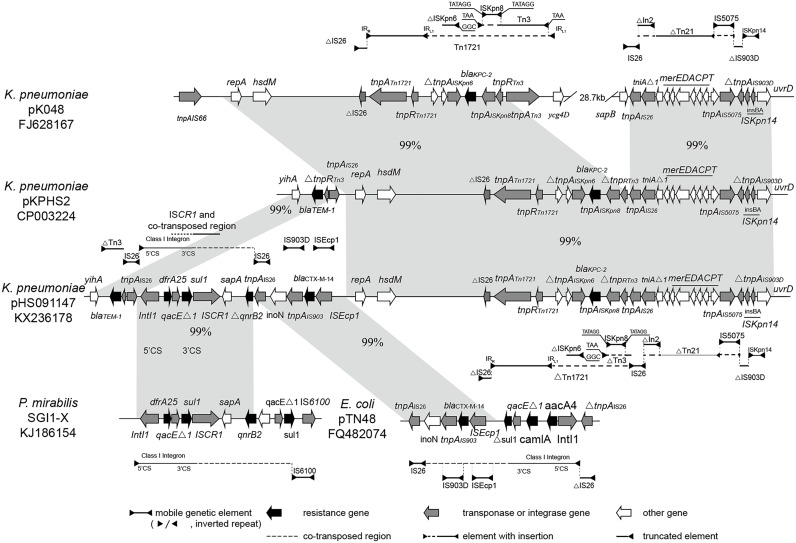
Comparative analysis of the multidrug-resistance region of plasmid pHS091147. The relevant parts of pKP048, pKPHS2, SGI1-X and pTN48 are shown to highlight the syntenic regions. The horizontal line shown above or below the schematics (with one or both ends demarcated by solid triangles to indicated inverted repeats) represents intact, interrupted or truncated ISs, transposons and integrons as appropriate. Interruptions in the structures above are indicated as dashed lines. Regions with similar sequences are indicated in gray with corresponding percentages between the plasmids.

The adaptation region, a continuous 49,218 bp IncFII_K2_ fragment, harbored five resistance genes (including *bla*_KPC-2_ and *dfrA25*), one truncated Δ*qnrB2* gene and some genetic elements like insertion sequences (ISs), transposons and integrons. It was highly mosaic-like and can be divided into three subregions that share strong homology with several different plasmids (Fig 3).

The first subregion, which co-harbored *bla*_KPC-2_ and *bla*_TEM-1_, showed >99% homology with *K*. *pneumoniae* plasmid pKPHS2 (GenBank accession number CP003224), which was also found in Huashan Hospital in 2011. Carbapenemase gene *bla*_KPC-2_ was located on a Tn*1721* transposon variant that was truncated by IS*26*, thus forming Tn*1721*-*bla*_KPC-2_-ΔTn*3*-IS*26*. It was the dominant *bla*_KPC-2_ genetic structure in forty-two non-duplicated, *bla*_KPC-2_-postive *K*. *pneumoniae* strains isolated in Huashan Hospital between August 2006 (when the first *bla*_KPC-2_-positive clinical *K*. *pneumoniae* isolate was detected) and October 2010 (when the *bla*_KPC-2_-positive isolates were detected continuously and steadily) [23]. It was fused, in turn, with the left end of ΔTn*21*-IS*5075*-ΔIS*903D*-IS*Kpn14*. Both the Tn*1721*-derived *bla*_KPC-2_-bearing transposon and the ΔTn*21*-IS*5075*-ΔIS*903D*-IS*Kpn14* contiguous region were highly similar to pKP048 (GenBank accession number FJ628167), a *K*. *pneumoniae* multidrug resistance plasmid carring *bla*_KPC-2_, *bla*_DHA-1_, *qnrB4*, and *armA* isolated in Zhejiang province of China. Given that the first subregion was highly related to pKP048, we inferred that this long segment derived from a pKP048-like plasmid following insertion of IS26 into the Tn*1721* variant. Subsequently, recombination mediated by the inserted IS*26* and the IS*26* adjacent to ΔTn*21* occurred and the intervening 35.3 kb fragment was deleted as a consequence [24]. The genetic environment of *bla*_TEM-1_ was consistent with the IS*26*-*bla*_TEM_ configuration reported in Australia in 2011 and located in a distance of 14kb from the *bla*_KPC-2_ fragment [25]. The other two subregions were located in-between.

The second subregion, IntI*1*-*dfrA25*-*sul1*-IS*CR1*-Δ*qnrB2*, shared >99% identity with the MDR region of the *Salmonella* genomic island, SGI1-X in *P*. *mirabilis* PmC162 (GenBank accession number KJ186154). The In*207* class 1 integron carried 5’-CS, which contained the *int1*, *dfrA25-attC* gene cassettes array (confers trimethoprim resistance) and the conserved segment, 3’-CS (*qacΔ1* and *sul1Δ*). The 3’-CS segment was followed by IS*CR1*-Δ*qnrB2*; the *qnrB2* gene was truncated by IS*26* flanking the third subregion.

The third subregion exhibited >99% homology to an *E*. *coli* CTX-M-14-encoding plasmid, pTN48 (GenBank accession number FQ482074), which included *bla*_CTX-M-14_ and its environment (IS*903* and IS*Ecp1B*), IS*26* and *iroN*. The *bla*_CTX-M_ genes were usually located within adjacent IS*Ecp1* areas, which provide a promoter for resistance gene expression [26, 27]. Moreover, CTX-M-14 was a highly malleable β-lactamase with broad opportunity to evolve. Many novel CTX-M-type ESBLs variants derived from CTX-M-14-like β-lactamase genes, culminating in higher MIC values [28, 29]. The high prevalence of CTX-M-14-producing *Enterobacteriaceae* has recently been reported in different provinces of China, with incidences ranging from 28.2% to 48.4% [14, 15, 30, 31]. In addition, the third subregion included *iroN*, the outer membrane siderophore receptor gene identified in *Salmonella* spp. *iroN*, which mediated utilization of structurally-related catecholate siderophores, was critical for virulence of the *iroBCDEN* gene cluster [32, 33]. It cannot function alone, and must operate in association with other virulence genes [33].

In conclusion, we describe here the complete sequence of a novel putative multi-replicon plasmid (pHS091147) obtained from a multidrug-resistant *K*. *pneumoniae* ST11 isolate, which carried five resistance genes (including *bla*_KPC-2_, *bla*_CTX-M-14_, *bla*_TEM-1_, *sul1* and *dfrA25*). Structural analysis showed that pHS091147 was highly mosaic and composed of parts previously identified in other plasmids of *Enterobacteriaceae* origin, suggesting that homologous recombination and horizontal gene transmission mediated by mobilizable elements played critical roles in evolution of the plasmid. The identified genes mediate resistance to last-line antimicrobial agents (carbapenems) and other, older generation antibiotics (i.e., trimethoprim). The location of these genes together on a single plasmid poses a serious epidemiological, clinical and public health threat.
